# Fabrication of SrTiO_3_ Layer on Pt Electrode for Label-Free Capacitive Biosensors

**DOI:** 10.3390/bios8010026

**Published:** 2018-03-16

**Authors:** Francesca Malvano, Luigi Maritato, Giovanni Carapella, Pasquale Orgiani, Roberto Pilloton, Marisa Di Matteo, Donatella Albanese

**Affiliations:** 1 Department of Industrial Engineering, University of Salerno, 84084 Fisciano, Italy; fmalvano@unisa.it (F.M.); lmaritato@unisa.it (L.M.); mdimatteo@unisa.it (M.D.M.); 2 Department of Physics “ER Caianiello”, University of Salerno, 84084 Fisciano, Italy; giocar@sa.infn.it; 3 Institute of Superconducting oxides and other innovative materials and devices of the National Council of Research (CNR), Unit Operative of Salerno, 84084 Fisciano, Italy; pasquale.orgiani@spin.cnr.it; 4 Institute of Crystallography of the National Council of Research (CNR), 00015 Monterotondo Scalo, Italy; roberto.pilloton@cnr.it

**Keywords:** biosensors, label-free, perovskite, strontium titanium oxide

## Abstract

Due to their interesting ferroelectric, conductive and dielectric properties, in recent years, perovskite-structured materials have begun to attract increasing interest in the biosensing field. In this study, a strontium titanate perovskite layer (SrTiO_3_) has been synthesized on a platinum electrode and exploited for the development of an impedimetric label-free immunosensor for *Escherichia coli* O157:H7 detection. The electrochemical characterization of the perovskite-modified electrode during the construction of the immunosensor, as well as after the interaction with different *E. coli* O157:H7 concentrations, showed a reproducible decrease of the total capacitance of the system that was used for the analytical characterization of the immunosensor. Under optimized conditions, the capacitive immunosensor showed a linear relationship from to 1 to 7 log cfu/mL with a low detection limit of 1 log cfu/mL. Moreover, the atomic force microscopy (AFM) technique underlined the increase in roughness of the SrTiO_3_-modified electrode surface after antibody immobilization, as well as the effective presence of cells with the typical size of *E. coli*.

## 1. Introduction

The impact of biosensing technology is increasing in all major sectors, such as pharmaceuticals, healthcare, environment, and food. Due to their simple use and application in relatively complex samples, biosensors offer a potential alternative to the most advanced bioanalytical systems [1]. The design of label-free affinity-based probing concepts is the objective of much current research, at both academic and industrial levels, towards establishing alternative methods to the already-existing enzyme-linked immunosorbent assay (ELISA)-based immunoassays. Among the different types of immunosensors that enable the direct monitoring of such interactions, impedimetric immunosensors have recently received particular attention since they possess a number of attractive characteristics associated with the use of electrochemical transducers, namely, the low cost of electrode mass production, cost effective instrumentation, the ability to be miniaturized and to be integrated into multi-array or microprocessor-controlled diagnostic tools, remote control of implanted sensors, etc. [2,3].

Most of the impedimetric immunosensors are fabricated by using, as the electrode, commercially very available gold or carbon materials (glassy carbon paste, nanotubes), which are cheaper, and easier to be fabricated than the first one, but discarded after use [4].

When taking measurements with such sensing electrodes, redox-active compounds are commonly added to the solution (faradaic approach), resulting in a well-defined charge transfer resistance, Rct.

However, if the layer on the electrode is an insulator the redox-active compound can be omitted resulting in a non-faradaic approach and a rather capacitive impedance behaviour will be observed. For this reason, when constructing a capacitive biosensor, the electrode surface is usually covered with an additional insulating layer and the capacitance change is induced when the dielectric constant [5] or the thickness of the double layer [6] on the transducer surface changes. Therefore, in contrast to the first faradaic scheme, no additional reagent is required for non-faradaic impedance spectroscopy, rendering non-faradaic schemes, usually measured at a single frequency, somewhat more available to different applications [7].

From the above, exploring new dielectric materials with high sensitivity and efficiency for the immobilization and detection of biomolecules is of importance in capacitive biosensor research.

In recent years, perovskite-structured materials and their derivatives have been widely studied in fundamental research for their high potential in technical applications because of their different physical properties: due to their attractive catalytic, dielectric, and photoelectric properties, perovskite oxides have been exploited as catalysts, semiconductors, electrodes, dielectric materials, luminescent materials, solar cells, and so on [8,9,10,11].

Perovskite-structured materials have begun to attract increasing interest in the biosensing field because their special properties, even if very few studies on biosensor fabrication based on perovskite materials have been reported in the literature [12,13,14].

To the best of our knowledge, perovskite-structured SrTiO_3_ have not been reported for biosensing applications so far. In this work, a strontium titanate perovskite layer (SrTiO_3_) has been synthesized on a platinum electrode and exploited for the construction an impedimetric label-free immunosensor for *Escherichia coli* O157:H7 detection. The immobilization of monoclonal *E. coli* antibody on the SrTiO_3_ layer was carried out via (3-Aminopropyl)triethoxysilane and glutaraldehyde. Electrochemical impedance spectroscopy (EIS) was used to characterize each step of the electrode modification and explore the analytical performance of the immunosensor. Finally, a comparison between immunosensors developed with and without the perovskite layer was conducted.

## 2. Materials and Methods

### 2.1. Chemicals

Cysteamine (95%), glutaraldehyde solution (50% in H_2_O), potassium hexacyanoferrate(III) ([Fe(CN)_6_]^3−^, >99%), and (3-aminopropyl)triethoxysilane (APTES, 99%) were purchased from Sigma-Aldrich (Milano, Italy). Potassium ferrocyanide ([Fe(CN)_6_]^4−^), was obtained from Carlo Erba reagent (Milano, Italy). Ethanolamine (EtNH_2_–NH_2_CH_2_CH_2_OH, >99.5%) was obtained from BioVision Inc. (San Francisco, CA, USA).

The *E. coli* O157:H7 antibody (1.4 mg/mL) was purchased from Fitzgerald (Acton, MA, USA), while the *E. coli* O157:H7 (heat-killed) was received from SeraCare (Gaithersburg, MD, USA).

Sodium phosphate monobasic (NaH_2_PO_4_), sodium phosphate dibasic anhydrous (Na_2_HPO_4_), and potassium chloride (KCl) used in the preparation of phosphate-buffered saline (PBS: 0.1 M KCl, pH 7.4) were obtained from Sigma Aldrich (Milano, Italy).

### 2.2. Apparatus

The electrochemical measurements were carried out with a computer-controlled Autolab PGSTAT 204 Potentiostat (Metrohm, Herisau, Switzerland), equipped with an impedance module (FRA32M); the experimental data were analysed with Nova software (Metrohm).

Thin-film single-electrodes, put in an all-in-one electrochemical cell, incorporate a conventional three-electrode layout, with platinum working, reference, and counter electrodes (Micrux Technologies, Oviedo, Spain). The diameter of the Pt working electrode was 1 mm.

### 2.3. SrTiO_*3*_ Perovskite Layer Deposition

SrTiO_3_ thin films were grown by the pulsed laser deposition technique using a KrF excimer pulsed laser source (=248 nm), with an energy density of about 4 J/cm^2^, under an ultra-pure (99.9999%) O_2_ pressure. The laser beam was focused on a stoichiometric SrTiO_3_ single-crystal and the high intensity energy of pulses allowed the congruent ablation of the target (APL 96, 032501 (2010)). In order to lower the deposition temperature as much as possible, it was varied from 750 °C down to 400 °C. Optimization of the deposition process was made by growing samples on (LaAlO_3_)_0.3_(Sr_2_TaAlO_6_)_0.7_ (LSAT) (100)-oriented single crystals. The temperature of the substrate was, therefore, set at 500 °C at a deposition pressure of 10–3 mbar. After the film growth, the samples were cooled to room temperature in about 30 min at deposition pressure. The typical deposition rate was about 0.05 nm per laser shot and the film thickness was set to about 100 nm.

**Figure 1:**
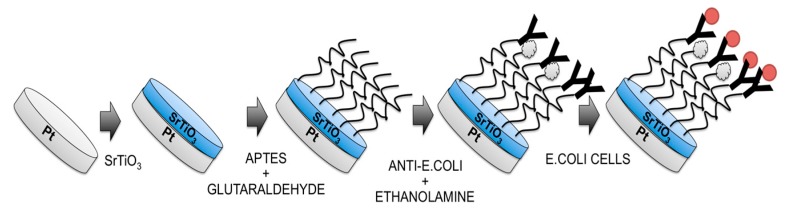
Steps used for the fabrication of *Escherichia coli* O157:H7 label-free immunosensor. At the end (right side) the measurement step is reported, too.

Then, by means of optical lithography and wet etching, the layer was selectively removed, using 7% HF in water as the selective etchant, from all substrate areas, except from the Pt electrode working area. In the final step only the working Pt electrode with a 1 mm diameter was covered by the SrTiO_3_ (see Figure 1).

### 2.4. Immunosensor Manufacturing

The SrTiO_3_-modified electrode was immersed in an ethanol solution containing APTES 10% (*v*/*v*) and sonicated for 20 min. After that, the 5% (*v*/*v*) glutaraldehyde solution was dropped onto the modified working electrode for 1 h and, again, the electrode was rinsed with water. Thereafter, the modified electrode was covered with 10 μL of anti-*E. coli* solution (1.4 ng/mL) for 30 min at room temperature. Finally, the unreacted active sites were blocked with 1 M ethanolamine and the electrode was rinsed in PBS to remove unbound antibodies.

The schematic diagram of immunosensor fabrication is shown in Figure 1.

The *E.Coli* immunosensor developed without the use of perovskite layer has been constructed as follows: Au electrode was immersed in cysteamine ethanol solution 20 mM and left overnight; after that, the electrode was thoroughly rinsed with water to remove physically-adsorbed cysteamine. Glutaraldehyde solution 5% (*v*/*v*) was dropped onto the cysteamine modified working electrode for 1 h and again the electrode was rinsed with water. Finally, the modified electrode was covered with 10 μL of anti-*E. coli* solution (1.4 ng/mL) for 30 min at room temperature; the unreacted active sites were blocked with 1 M ethanolamine and the electrode was rinsed in PBS to remove unbound antibodies.

### 2.5. Experimental Measurements

Electrochemical impedance spectroscopy (EIS) was used to characterize each step of the electrode modification. EIS measures the response of an electrochemical system to an applied oscillating potential as a function of the frequency resulting in an impedance spectra (Nyquist plot) where the complex impedance is displayed as the sum of the real and imaginary components (Z′ and Z″, respectively). In particular, a sinusoidal AC potential (10 mV) in the frequency range from 0.1 to 10^5^ Hz was superimposed to 0.00 mV (vs. the reference electrode) DC potential.

The measurements were performed in phosphate buffer, as background electrolyte, at room temperature.

For the *Escherichia coli* O157:H7 measurement, 1 mL of diluted heat-killed *E*. *coli* O157:H7 cells, ranging in concentration from 1 to 10^6^ cells/mL, was dropped onto the electrode working area and incubated for 90 min; before the impedance measurements the immunosensor was rinsed thoroughly with copious amounts of bi-distilled water.

## 3. Results

### 3.1. Characterization of the Electrode Modification Process

An electrode immersed in an electrolyte solution can generally be described as resembling a capacitor in its ability to store charge. Furthermore, when the surface of the electrode is completely covered by a dielectric layer, the whole electrode behaves as an insulator [15].

**Figure 2:**
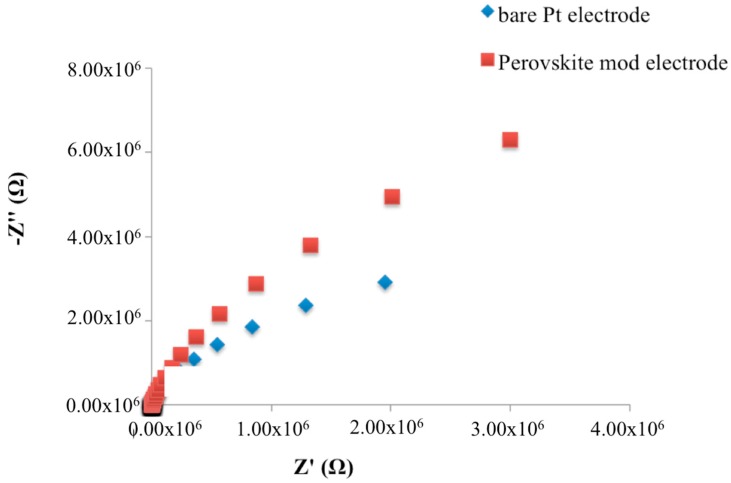
Nyquist plots in non-faradaic impedance measurements of bare and SrTiO_3_-modified Pt electrodes.

Therefore, as expected, the experimental complex plane impedance spectra for the bare Pt electrode and SrTiO_3_ perovskite layer modified Pt electrode, obtained under non-faradaic conditions, exhibited an almost linear and close-to-vertical spectra (Figure 2), which indicates a purely capacitive response of the electrode properties [16,17]. In particular, when the SrTiO_3_ perovskite layer has been deposed, an increase of the curve slope was observed.

In order to characterize the behaviour of the sensing layers needed for receptor immobilization, EIS measurements have been carried out during each step of the immunosensor construction.

**Figure 3:**
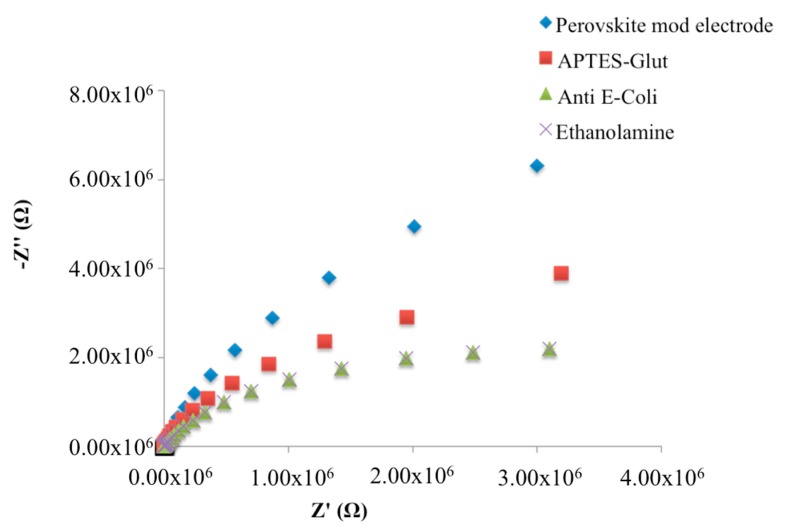
Nyquist plots in non-faradaic impedance measurements during the fabrication of the immunosensor.

The APTES layer, as well as the consecutive immobilization of monoclonal antibodies, on SrTiO_3_ layer, caused a decrease of the impedimetric curve slope in Nyquist plots, which represent the change of the two impedance components as a function of the frequency (Figure 3).

No impedimetric changes were observed after ethanolamine deposition.

When constructing a capacitive biosensor, where the second plate is represented by the electrolyte, changes in the dielectric properties and/or thickness of the dielectric layer at the electrode-electrolyte interface are exploited [3]. The electric capacitance between the working electrode and the electrolyte can be described by Equation (1):

(1) $$C=\frac{\varepsilon_{0}\varepsilon A}{d}$$

where *ε* represents the dielectric constant of the medium between the plates, *ε*_0_ is the permittivity of free space, *A* is the surface area of the plates and *d* is the thickness of the insulating layer [5]. Thus, when there is a change in the dielectric properties in the material between the plates, a change in the capacitance will occur [5].

In the Nyquist plots obtained during the fabrication of immunosensor (Figure 3) we observe a decrease in the imaginary component (−Z″) of total impedance at low frequencies, which is inversely proportional to the capacitance of the system [3]. Thus, an increase of the total capacitance due to the change in dielectric behaviour of the immobilized molecules is, thus, observed upon the binding of the analyte to its specific receptor.

### 3.2. AFM Characterization

In order to obtain information about the morphological characteristics of electrode surface at different steps of immunosensor construction, atomic force microscopy (AFM) imaging was used. The AFM scans were carried out in the low-amplitude intermittent contact (tapping) mode, in air.

**Figure 4:**
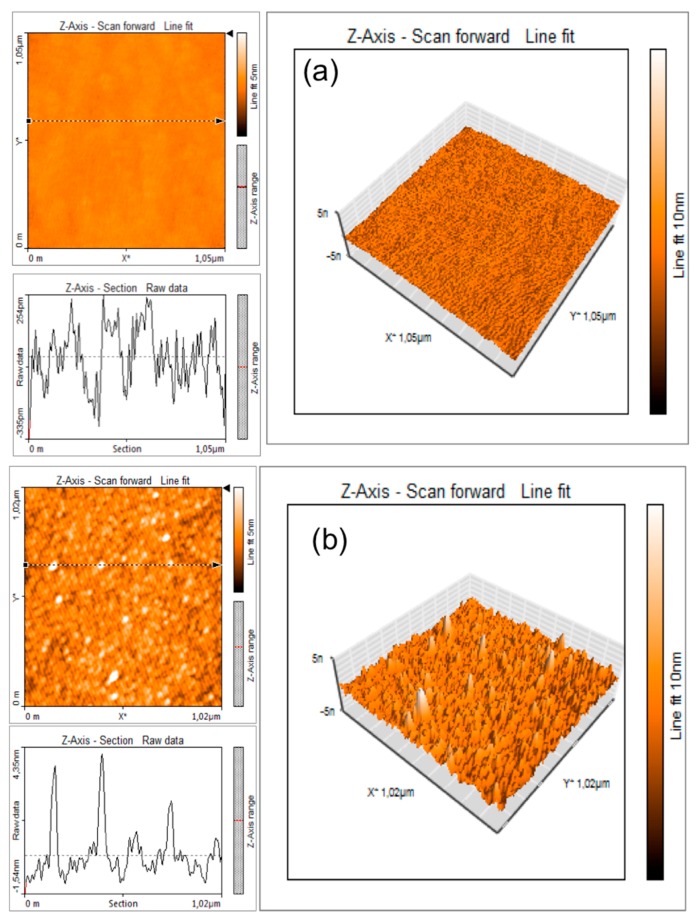
Roughness of the SrTiO_3_-modified electrode surface before (**a**) and after (**b**) antibody immobilization.

Figure 4 shows the change in roughness of the electrode surface before (Figure 4a) and after (Figure 4b) antibody immobilization.

As expected, starting from a very low peak-to-valley line roughness (R_y_ below 1 nm) of the SrTiO_3_-modified electrode (Figure 4a), after the functionalization with anti-*E. coli*, the peak to valley line roughness increases to R_y_ = 5 nm due to the presence of nanostructures with a typical height of 4–6 nm and a diameter of 30–40 nm. These figures, accounting for the finite curvature radius of the AFM tip (10 nm), are compatible [18] with the apparent dimensions of an antibody.

**Figure 5:**
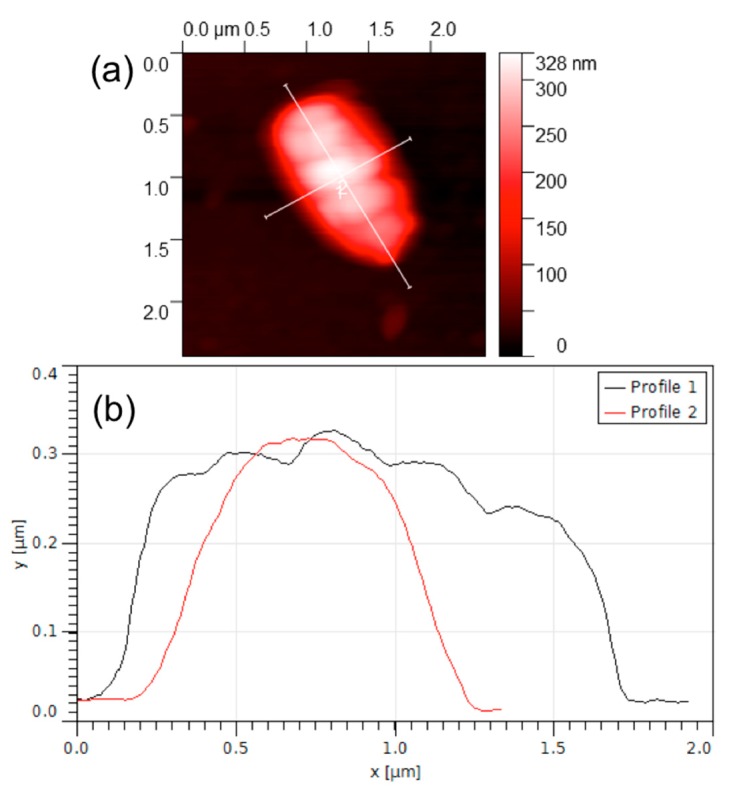
AFM image of an *E. coli* cell. (**a)** AFM image (**b**) observational results.

Figure 5 shows an AFM image of a selected working area functionalized with *E. coli* antibodies and exposed to 6 log cfu/mL of *E. coli* solution.

A micro-object of approximately 1.8 μm × 1 μm × 0.3 μm can clearly be seen in this picture. The dimensions of the micro-object correspond to the common dimensions of an *E. coli* bacterium that is known to be a cylindrical object, measuring approximately 2 μm in length and 1 μm in diameter [19,20]. As reported previously [20], the slightly reduced dimensions of the bacterial cell observed in this experiment could be explained by the possible flattening occurring due to the pressure exercised by the AFM cantilever tip as well as shrinking of the bacteria due to the drying and fixing procedure.

### 3.3. Analytical Parameters of an E. coli Capacitive Label-Free Immunosensor

**Figure 6:**
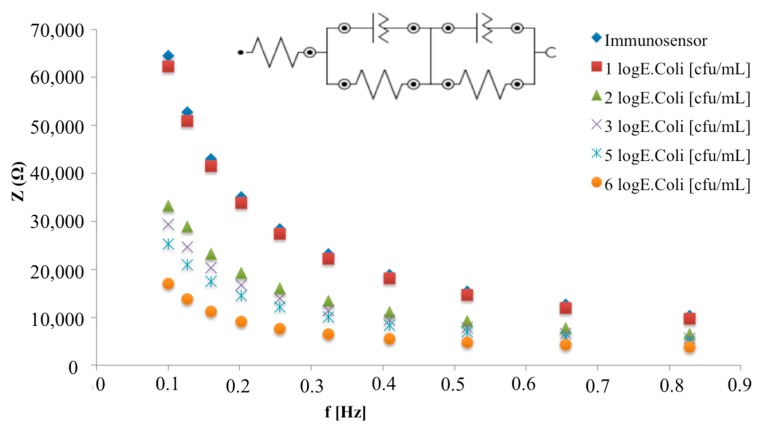
Bode plots in non-faradaic impedance measurements of the immunosensor before and after the interaction with different *E. coli* concentrations in the frequency range 0.1–1 Hz. The inset shows the equivalent circuit used for impedimetric data fitting.

The developed immunosensor was incubated with increasing concentrations of *E. coli* cells and the Bode plots report the total impedance as a function of frequency: while no differences are shown in the higher frequency region (inset Figure 6), significant total impedance changes are shown from 0.1 to 1 Hz (Figure 6). In particular, a decrease of the total impedance was observed with increasing *E. coli* concentration.

A metal electrode modified with a recognition layer can be describe as a capacitor when placed in an electrolyte solution. In the non-faradaic approach, the total capacitance can be described as a combination of two capacitors in series: the first one corresponds to the dielectric layer on the working surface (*C_dl_*) and the second one corresponds to the biomolecule layer (*C_bm_*) that includes the anchoring group, the bound recognition element and any contribution from the Stern layer (which consist of hydrophobically-bound water molecules between the recognition and the diffuse layer) and, consequently, the interaction of this layer with specific ligand. In this case, the circuit commonly used for the impedimetric data fitting consists of series capacitive elements, and the total capacitance *C_t_* can be described as follows [3]:

(2) $$\frac{1}{C_{t}}=\frac{1}{C_{dl}}+\frac{1}{C_{bm}}$$

This model implies an ideal dielectric behaviour of the insulating and biomolecule layers, which commonly reveals, in a Nyquist plot, a straight line parallel to the imaginary impedance axis. However, this situation is not common for the electrolytic capacitors: defects in the construction of the insulating layer (pin-holes) are the mayor reason for the non-ideal dielectric behaviour of an assembly.

In this case, the equivalent circuit which better fit impedimetric data includes a resistance as a parallel element to each of the corresponding capacitors, which represent the resistance of the insulating layer to species moving through the collapse sites and pin-holes within the structures [21].

Based on the above, impedimetric data obtained for the increasing *E. coli* concentrations were fitted with the equivalent circuit showed in the inset of Figure 6, and the total capacitance of the system was calculated according to Equation (2).

According to Supaporn [22], from the fit of experimental data a decrease in capacitance from 0.196 nF/cm^2^ for 1 log cfu/mL *E. coli* cells to 0.007 nF/cm^2^ for 7 log cfu/mL *E. coli* cells is highlighted.

Throughout the whole study, the percentage change in capacitance (denoted as *ΔC*%), taken as a measure before and after the immunoreaction, was used to study the analytical parameters of the developed immunosensors; it was calculated by the following equation:

(3) $$∆C\%=\frac{\left(C_{antiE.Coli}-C_{antiE.Coli-E.Coli}\right)}{C_{antiE.Coli}}\times 100$$

where $C_{antiE.Coli-E.Coli}$ is the value of capacitance after immunoreaction and $C_{antiE.Coli}$ represents the value of the capacitance of the native immunosensor.

**Figure 7:**
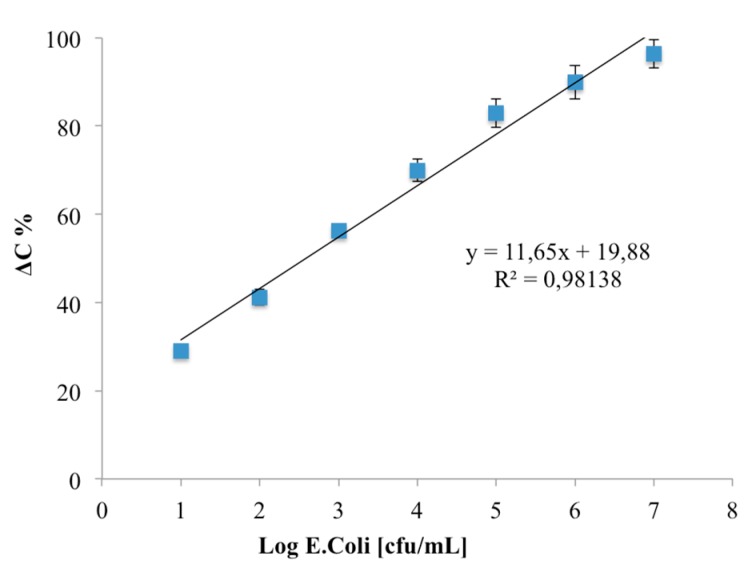
Calibration curve of *Escherichia coli* O157:H7 immunosensor. Data represent the average values of five immunosensors.

The calibration curve (Figure 7) shows a linear correlation between *ΔC* and log *E. coli* (cfu/mL) in the range from 1–7 log cfu/mL: no linear *ΔC* was observed due to the saturation of the specific binding sites at higher concentration. The limit of detection, based on the sum of the average blank solution and three times the standard deviation, was estimated to be 1 log cfu/mL.

**Figure 8:**
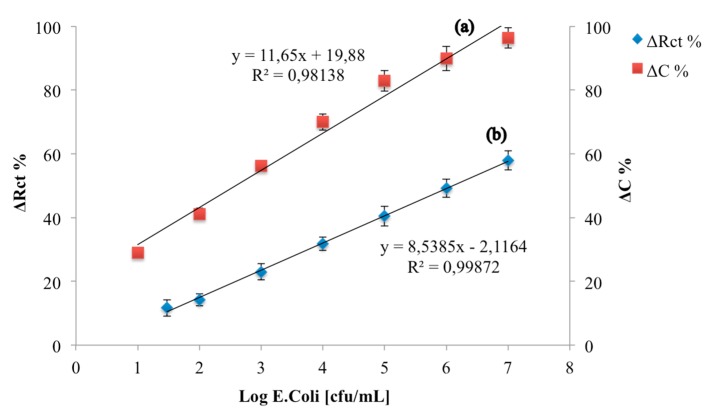
Calibration curves of a capacitive *E. coli* immunosensor (**a**) and a resistive *E. coli* immunosensor (**b**).

Finally, the analytical parameters of the developed immunosensor were compared with those obtained with an immunosensors developed on a Au electrode, on which the same quantity of monoclonal *E. coli* O157:H7 antibody has been immobilized using cysteamine monolayer and GA (Figure 8). The analytical performances of this immunosensor were characterized under faradaic conditions according to [23,24].

The calibration curves highlight that the percentage variation of the capacitive component is greater than the resistive one, thus making the capacitive immunosensor more sensitive than the resistive one and able to detect a lower limit of detection (1 log cfu/mL for capacitive immunosensor, 1.47 log cfu/mL for resistive immunosensor).

Moreover, both immunosensors showed high reproducibility calculated on five *E. coli* immunosensors at *E. coli* 3 log cfu/mL (equal to 3.1% for the capacitive immunosensor and 3.9% for the resistive one). Table 1 shows the analytical parameters of both of the developed immunosensors.

The comparison of the analytical performances of the capacitive label-free immunosensor developed in this study with the other impedimetric label-free immunosensors in the literature is reported in Table 2.

The capacitive immunosensor developed with antibodies immobilized on a SrTiO_3_ perovskite layer showed a lower LOD when compared with the other impedimetric label-free immunosensors for *E. coli* detection.

## 4. Conclusions

Exploiting the well-known dielectric properties of perovskite, an innovative label-free capacitive immunosensor for the pathogenic strain (O157:H7) of *E. coli* was developed.

The proposed capacitive immunosensor, with its very low detection limit (1 log cfu/mL) and high sensitivity, underlines the attractive characteristics associated to the use of SrTiO_3_ for sensing applications. The first results obtained in this study underline the technologically-promising properties of SrTiO_3_ perovskite, including conductive and dielectric characteristics, making it fascinating for wide applications in electrochemical sensing.

## Figures and Tables

**Table 1:** Analytical parameters of capacitive and resistive *E. coli* immunosensors developed in this work.

Working Principle	Sensitivity (% Change)	Linear Range (log cfu/mL)	LOD (log cfu/mL)	RSD (%)
Capacitive	11.65	1–7	1	3.1
Resistive	8.53	1.47–7	1.47	3.9

**Table 2:** Comparison among impedimetric label-free *Escherichia coli* O157:H7 immunosensors.

Schematic Immunosensor Assembly	Working Principle	Linear Range (log cfu/mL)	LOD (cfu/mL)	References
Au + PANI + Glu + anti-*E. coli*	Resistive	2–7	100	[25]
Au + MHDA	Resistive	1.47–8.48	2	[26]
GOPE + AuNPs + Streptavidn + Biotin + anti-*E. coli*	Resistive	2.17–8.17	150	[27]
Epoxysilane-mod. ITO + PDMS + anti-*E.* *coli*	Resistive	1–6	10	[28]
Pt + SrTiO_3_ + APTES-Glu + anti-*E. coli*	Capacitive	1–7	10	This work
Au + Cys + Glu + anti-*E. coli*	Resistive	1.47–1	30	This work

PANI: polyaniline; Glu: glutaraldheyde; MHDA: 16-mercaptohexadecanoic acid; GOPE: graphene oxide paper electrode; PDMS: poly(dimethylsiloxane).
